# When Autoimmunity and Vascular Events Collide: A Unique Presentation of Hashimoto’s Encephalopathy and Ischemic Stroke

**DOI:** 10.7759/cureus.98423

**Published:** 2025-12-03

**Authors:** Marilhia C Cornejo Leon, Nariman Noorbakhsh-Sabet

**Affiliations:** 1 Department of Neurology, The University of Tennessee Health Science Center (UTHSC), Memphis, USA

**Keywords:** autoimmune encephalopathy, autoimmune thyroid disorder, hashimoto’s encephalopathy, ischemic stroke, watershed stroke

## Abstract

Hashimoto’s encephalopathy (HE) encompasses an umbrella of symptoms, including cognitive decline, seizures, neurological focal deficits, stroke, psychosis, dementia, and coma. Its pathogenesis is not clear, nor is its mechanism by which it causes strokes.

A 56-year-old African American female with nodular thyroid disease presented with subacute encephalopathy, amnesia, and bilateral symmetric cerebral watershed strokes in the setting of elevated antithyroid antibodies that clinically improved after immunotherapy. This is a rare reported case of possible autoimmune thyroiditis (AT) presenting with watershed strokes.

Thyroid disorders are a risk factor for cerebrovascular disease. Autoimmune thyroiditis increases the risk of stroke in patients with hypothyroid AT due to the higher frequency of atrial fibrillation, large artery atherosclerosis, and hypertension. The pathogenesis of HE is uncertain, but it is believed that it could be autoimmune, vasculitis, and/or hypoperfusion-related. Our patient had three episodes of hypotension, raising concern for global cerebral hypoperfusion due to the microvascular disruption that takes place in HE.

HE has a low prevalence but good response to steroids. Considering it as a differential diagnosis in cases of thyroid disease and acute stroke is crucial to promptly make a diagnosis and start treatment.

## Introduction

Hashimoto’s encephalopathy (HE) is an infrequent disease that can present with many of the common symptoms seen in hospital consultations. Learning about HE allows clinicians to consider it in the differential diagnosis of patients with cognitive decline, seizures, strokes, neurological focal deficits, dementia, and coma [1,2]. HE was initially described in 1966 as an acute or subacute encephalopathy or myelopathy associated with high serum levels of antithyroid antibodies (anti-thyroglobulin antibodies (anti-TG) and antithyroid peroxidase antibodies (anti-TPO)), normal or abnormal thyroid function, and good response to corticosteroids [1].

The pathogenesis of HE is not fully understood, but evidence shows a possible autoimmune pathogenesis. An association with Hashimoto thyroiditis (HT) has been hypothesized [3]. However, research has shown that there is no correlation between disease activity and the titers of antibodies. Also, antithyroid antibodies are not specific indicators, as they may be elevated in 10% of the general population (anti-TPO at 11.3% and anti-TG at 10.4%) [4].

Another highlight of HE is that it is a classical example of a stroke chameleon. The mechanism by which HE causes stroke is not clear; case reports of HE have described strokes caused by autoimmune vasculitis [5,6], carotid atherosclerosis, atrial fibrillation, and hypoperfusion [7,8].

We report a case of encephalopathy, amnesia, and bilateral symmetric cerebral watershed strokes in the setting of elevated antithyroid antibodies, which clinically improved after immunotherapy. As far as we know, this is a rare reported case of possible autoimmune thyroiditis (AT) presenting with watershed strokes.

## Case presentation

**Figure 1:**
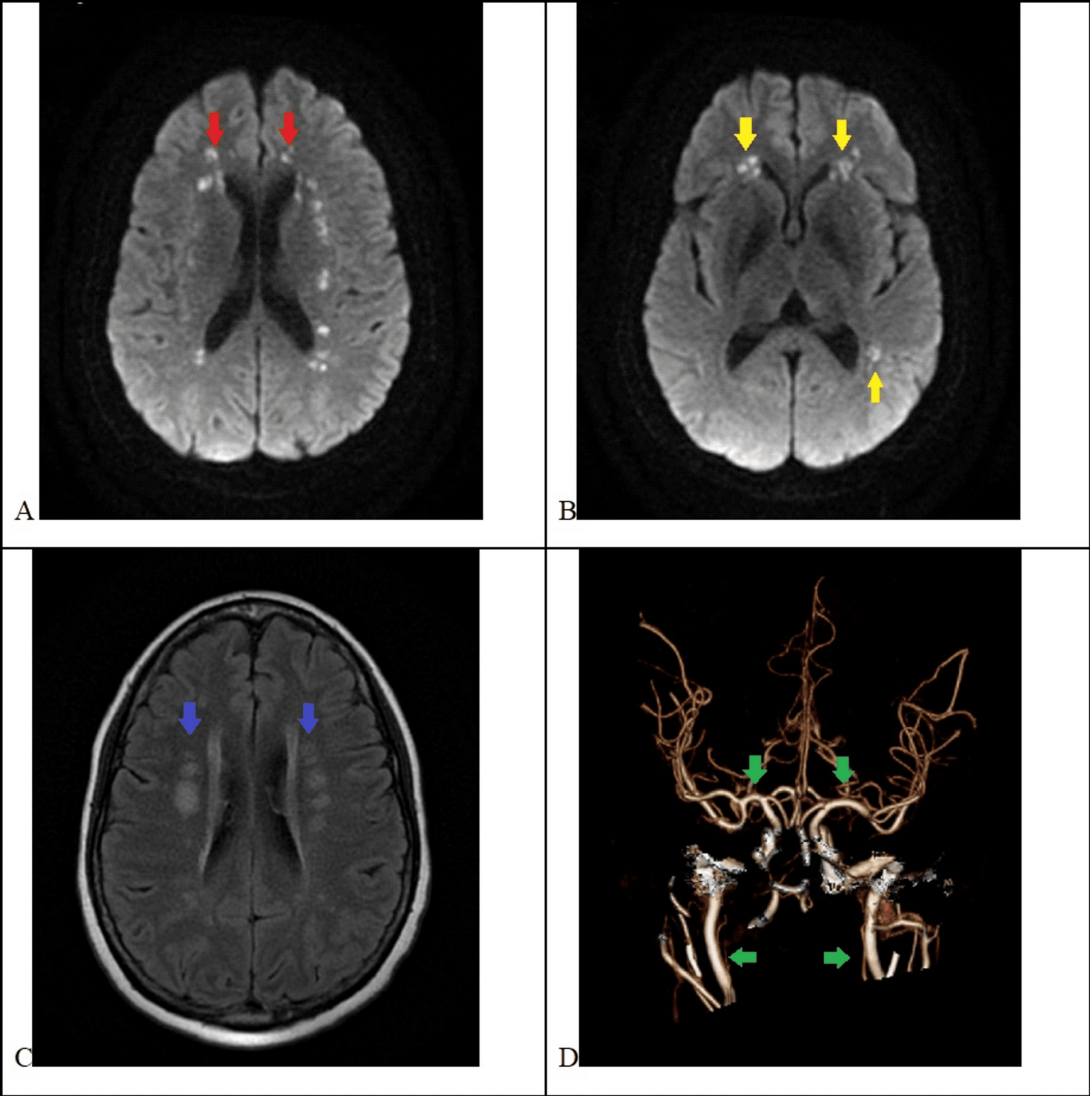
Brain magnetic resonance imaging with bilateral cortical and subcortical watershed strokes (A, B) MRI diffusion-weighted imaging (DWI) showing bilateral acute cortical (red arrows) and subcortical (yellow arrows) watershed strokes. (C) MRI T2 fluid-attenuated inversion recovery (FLAIR) showing bilateral subcortical watershed strokes (blue arrows). (D) CT angiogram of the head showing the circle of Willis with no high-grade stenosis in the anterior circulation (green arrows).

A 56-year-old African American woman with a history of nodular thyroid disease (fine needle aspiration (FNA) biopsy benign), anxiety, and a recent hospital admission for diplopia and generalized weakness - suspected to be myasthenia gravis and treated with intravenous immunoglobulin (IVIG) with clinical improvement and negative antibodies (acetylcholine, anti-MuSK, anti-GQ1b IgG) - presented with an acute onset of decreased level of consciousness. On evaluation, the patient was somnolent, oriented to person, and unable to recall events of the past weeks. Cranial nerves were intact, and she was following simple commands intermittently. Her muscle strength was 3/5 in the proximal right upper extremity (RUE) and 4/5 in the other extremities. Reflexes were decreased in the bilateral lower extremities, and sensory examination was preserved. Computed tomography (CT) of the head and CT angiography were unremarkable, while magnetic resonance imaging (MRI) showed bilateral cortical and subcortical symmetric watershed strokes (Figure 1). There was no reported episode of hypotension or cardiac arrest on prior admission, but there were records of one vasovagal syncope event with a negative MRI brain. Telemetry was negative for arrhythmias.

Lumbar puncture showed 192 cells, 94% lymphocytes, glucose 56 mg/dL, and protein 24 mg/dL. Infectious, autoimmune, and paraneoplastic panels in serum and cerebrospinal fluid - including RPR (rapid plasma reagin), HIV, cryptococcal antigen, and fungal cultures - were negative. Thyroid hormones showed low TSH of 0.21 μIU/mL, with normal free T4 and free T3. Anti-thyroglobulin (anti-TGB) and anti-microsomal antibodies were elevated at 13.5 and 45.8 IU/mL, respectively. CRP and ESR were mildly elevated. Urine drug screening was positive for benzodiazepines, due to home medication of valium 10 mg prescribed as needed for anxiety. Spot and continuous EEG showed mild generalized slowing without seizures or epileptiform activity. The embolic workup hypercoagulation panel showed that antiphospholipid and anticardiolipin panels were normal; antithrombin III was elevated, and protein S was decreased. Transthoracic and esophageal cardiac ultrasounds were normal. The elevated antithyroid antibodies and CSF lymphocytic pleocytosis support the diagnosis of HE (Table 1).

**Table 1:** Laboratory results *Autoimmune and paraneoplastic CSF panel includes anti-Hu, anti-Ri, ANA, PCA, autoimmune encephalopathy, Purkinje cell cytoantibody, amphiphysin, CRMP5 IgG, AGNA 1, DPPX, mGluR1, IgLON5, Ma2/Ta, Zic4, DNER, ITPR1, AMPA R1 cell-based IFA, AMPA R2 cell-based IFA, GABAB receptor, NMDAR, GAD65, CASPR2, and LGI1. TSH: Thyroid-stimulating hormone; RPR: Rapid plasma reagin; HIV: Human immunodeficiency virus; CRP: C-reactive protein; ESR: Erythrocyte sedimentation rate; anti-TGB Ab: Anti-thyroglobulin antibody; ANA: Antinuclear antibody; ANCA: Antineutrophil cytoplasmic antibody; SSA: Anti-Sjögren's syndrome-related antigen A autoantibody; SSB: Anti-Sjögren's syndrome-related antigen B autoantibody; APC: Activated protein C; CSF: Cerebrospinal fluid; GABAB receptor: Gamma-aminobutyric acid B receptor antibody; NMDAR: N-methyl-D-aspartate receptor antibody.

Laboratory	Values	Reference values
Serologic tests		
Metabolic		
TSH	0.29	0.35-4.94
T4 free	1.10	0.70-1.48
T3 free	1.90	1.58-3.91
Infectious		
RPR	Negative	
HIV	Negative	
Inflammatory/Autoimmune		
CRP	9.3	<5
ESR	35	0-15
Anti-TGB Ab	13.5	<4
Anti-microsomal Ab	45.8	<5.6
Anti-MuSK IgG	<1	<1
Rheumatoid factor	<13	0-30
ANA panel	Negative	
ANCA panel	Negative	
Anti-Smith	<0.2	<1
Anti-RNP	<0.2	<1
Anti-SSA	1.1	<1
Anti-SSB	<0.2	<1
Hypercoagulable		
Anticardiolipin panel	<1.6	<20
Beta-2 glycoprotein	<1.4	<20
Antithrombin III	130	84-125
Protein S	38	47-134
Protein C	114	74-161
APC	2.3	≥2.1
CSF		
Nucleated cells	192	0-5
Lymphocytes	180 (94%)	0%-5%
Glucose	56	40-70
Protein	24	15-40
Biofire meningitis	Negative	
Autoimmune panel*	Negative	
Paraneoplastic panel*	Negative	
Cryptococcal antigen	Negative	
Fungal culture	Negative	
Urine tests		
Urine drug screen	Benzodiazepines	
Urinalysis	Negative	

The decreased level of consciousness did not correlate with the burden of stroke. The abnormal thyroid panel raised concern for HE. She received five daily doses of methylprednisolone 1 g in 50 cc of 0.9% sodium chloride, resulting in clinical resolution of her mental status and partial improvement of the RUE drift and amnesia. Of note, on both days following the course of steroids, she had an episode of hypotension with rapid recovery. As there were no new neurological deficits, a new MRI was not performed. She was discharged on prednisone tapering, midodrine, and follow-up appointments with endocrinology, rheumatology, and neurology clinics.

## Discussion

Thyroid disorders are a risk factor for cerebrovascular disease. Abnormal levels of TSH, with normal or abnormal peripheral thyroid hormone levels, are associated with stroke. Among the different ways stroke can occur, thyroid disease can cause cardioembolic strokes through hyperthyroidism-induced atrial fibrillation (AF) and large- and small-vessel disease strokes by hypothyroidism-induced hyperlipidemia, hypertension, diabetes, and obesity [9]. Moreover, thyroid autoimmune disorders can cause stroke by inducing atherosclerosis, atherothrombosis, and, more rarely, central nervous system vasculitis [9,10]. AT demonstrates a 10%-14% increased risk of stroke in patients with hypothyroid AT due to the higher frequency of AF, large artery atherosclerosis, and hypertension [11]. The pathogenesis of HE is uncertain, and contrary to expectations, the majority of patients are euthyroid at presentation. Therefore, it is believed that the pathogenesis is possibly autoimmune, vasculitic, and/or hypoperfusion-related [9,12,13].

Our patient’s clinical presentation meets the modified Peschen-Rosin criteria for HE, and she responded to high-dose steroids, correlating with the diagnosis of HE. Nevertheless, she received IVIG, which could contain antithyroid antibodies leading to false-positive results [14]. However, clearance occurs within 7-21 days, and the serum titer was collected on day 14 after the last IVIG dose.

Despite the concurrence of clinical symptoms and bilateral symmetric cortical and subcortical watershed strokes (Figure 1), we were not able to prove an association with HE, as she had neither extracranial nor intracranial significant vessel stenosis, and this stroke mechanism has never been reported to be associated with HE. Outside hospital records reported a sudden hypotensive event followed by a brief generalized jerking a day after completing the five-day IVIG course. Symptoms resolved quickly after fluids, and the EEG and brain MRI were negative. Her husband reported two episodes of symptomatic hypotension: one as described in the outside hospital record and another in the current admission, both occurring the day after she finished the steroid course.

Could the patient have had an unwitnessed hypotensive episode before arrival that explains the watershed strokes? If so, are these episodes related to HE or medication side effects? Studies have identified alpha-enolase, an autoantigen of the brain and thyroid, in the serum of HE patients [15]. This may imply that HE pathogenesis is either due to vasculitis, as alpha-enolase has been described in other autoimmune vasculitides such as SLE, ANCA, and RA, or due to global cerebral hypoperfusion, as a consequence of disruption of the brain endothelium, where alpha-enolase is highly expressed [15]. We hypothesize that the distribution and characteristics of the stroke are secondary to hypoperfusion, which could be a result of dysregulation of blood pressure due to microvascular disruption of HE.

Given the IVIG clouding the interpretation of antithyroid antibody titers, endocrinology will reassess them at the 30-day follow-up. The decision regarding whether to require a digital subtraction angiography and/or brain biopsy will be made as an outpatient, depending on the course of the disease.

## Conclusions

The mechanism of HE-related strokes is undetermined. In cases of HE with hyper- or hypothyroid AT, the main stroke risk factors are induced AF and large artery atherosclerosis. However, in cases of euthyroid HE, autoimmune vasculitis and cerebral hypoperfusion are possibly the most likely etiologies. Moreover, as the symptoms are very nonspecific, it is important to perform a thorough workup to exclude other metabolic, infectious, inflammatory, and neoplastic diseases. The low prevalence of HE limits the ability to achieve conclusive data, but it has a good response to steroids; therefore, considering HE in the differential and obtaining a prompt diagnosis is crucial.
